# Evaluation of quality and antimicrobial efficacy of locally manufactured alcohol-based hand sanitizers marketed in Addis Ababa, Ethiopia in the era of COVID-19

**DOI:** 10.1186/s13756-022-01163-2

**Published:** 2022-10-08

**Authors:** Muluken Nigatu Selam, Bruck Messele Habte, Tesfa Marew, Molalegne Bitew, Tigist Getachew, Solomon Getachew, Atlaw Abate, Mequanint Mitiku, Motlalepula Matsabisa, Gebremariam Birhanu

**Affiliations:** 1 Department of Pharmaceutics and Social Pharmacy, School of Pharmacy, College of Health Sciences, Addis Ababa University, P.O. Box 1176, Addis Ababa, Ethiopia; 2 Bio and Emerging Technology Institute, Addis Ababa, Ethiopia; 3 Ethiopian Food and Drug Authority, Addis Ababa, Ethiopia; 4 Department of Medical Laboratory, Tikur Anbessa Specialized Hospital, College of Health Sciences, Addis Ababa University, Addis Ababa, Ethiopia; 5 Department of Pharmacology, Faculty of Health Sciences, University of the Free State, Bloemfontein, 9300 South Africa

**Keywords:** Antimicrobial efficacy, COVID-19, Alcohol-based hand sanitizer, Quality evaluation

## Abstract

**Background:**

The coronavirus disease 2019 (COVID-19) has been rapidly spreading across the globe since the World Health Organization (WHO) has declared the disease outbreak as a global pandemic on March 11, 2020. Hand hygiene, via either regular handwashing with soap and water or using hand sanitizers, is among the various measures that need to be followed to control the outbreak of the disease. Alcohol-based hand sanitizers (ABHS) are the “gold standard” for hand disinfection because of their broad antimicrobial spectrum of activity, easy availability, better safety profile, and general acceptability to users. This study aimed at evaluating the physicochemical quality and antimicrobial efficacy of the locally manufactured ABHS marketed in Addis Ababa, Ethiopia.

**Methods:**

A cross-sectional survey was used to collect ABHS from Addis Ababa marketplaces. A total of 25 sample products were randomly selected from different categories of hand sanitizer manufacturers. The physicochemical evaluation of the products was carried out as per the United States Pharmacopoeia and WHO standards. Escherichia coli, Klebsiella spp., Pseudomonas aeruginosa, Staphylococcus aureus, Salmonella spp., and Shigella spp clinical isolates were used for the antimicrobial efficacy test.

**Results:**

The Fourier Transform Infrared result confirmed that all the test products met the identification test for ethanol. The majority (68%) of ABHS complied with the test for ethanol content (75–85% v/v). However, only 3 products fulfilled the hydrogen peroxide content (0.112–0.137% v/v). LPC307 showed the maximum zone of inhibition of 12 mm against Escherichia coli whereas MPC204 exhibited only 3 mm. LPC101 was found to be more sensitive to Shigella and Klebsiella Spp with minimum inhibitory concentration values of 20% and 10%, respectively. The sample product LPC101 showed a minimum bactericidal concentration of 20% against Escherichia coli, Pseudomonas aeruginosa, and Klebsiella spp.

**Conclusion:**

One-third of the tested ABHS did not comply with the WHO ethanol content limit and the majority of the products failed to meet the label claim for hydrogen peroxide content. Besides, nearly all products proved that they have activity against all the tested pathogenic microorganisms at a minimum concentration from 10 to 80%; though, they did not show 99.9% bacteriostatic or bactericidal activities as claimed. The study findings suggested regular monitoring of the quality of marketed ABHS considering the current wide use of these products.

## Introduction

Coronavirus disease 2019 (COVID-19) is continuing to spread around the world, with above 493 million confirmed cases and more than six million deaths affecting over 200 countries worldwide as of April 07, 2022 [1]. This highly contagious viral illness is caused by severe acute respiratory syndrome coronavirus 2 (SARS-CoV-2) (2). COVID-19 is emerging as the most consequential global health crisis since the era of the influenza pandemic of 1918 [2]. In Ethiopia, the total number of infections and deaths due to the COVID-19 pandemic is 469,916 and 7,508, respectively as of April 07, 2022 [1].

Keeping the cleanliness of hands is among the various measures that need to be followed to control the spread of COVID-19 and other infectious diseases which can be affected via either regular handwashing with soap and water or using hand sanitizers [3–5].

Out of the various commercialized hand sanitizer products, the most popular and demanding formulations are alcohol-based hand sanitizers (ABHS) containing ethanol as an active ingredient [3, 6]. World Health Organization (WHO) strongly recommends the use of ABHS, which is regarded as the “gold standard” for hand disinfection in healthcare facilities in the community because of its broad antimicrobial spectrum of activity on various microbial strains. Currently, hand sanitizers are habitually used for disinfection against SARS-CoV-2 dueto their easy availability at the point of care, better safety profile, and general acceptability to users [6–9]. In addition, the use of ABHS for protection against bacteria (gram-positive and negative), mycobacteria, fungi, and viruses is well documented [10–12].

As a standard of care for hand hygiene practice, WHO recommended use of ABHS containing either ethanol or isopropyl alcohol at strengths of 80% or 75% v/v, respectively [13]. Hands rubbing using ABHS for 25–30 s is reported to kill 99.99% of microorganisms on hands [14, 15].^.^ Hence the strength of the formulations should be evaluated as the alcohol concentration is an active agent and a critical determinant of ABHS efficacy [16].

Along with the increasing demand, the number of sanitizer manufacturers is booming making quality assurance and regulatory functions complicated. It is fact that the effectiveness of the ABHS is highly dependent on their quality and proper use. The high demand for such affordable products could have made them a candidate for counterfeiting [17].

Hand sanitizers are regulated as Over-the-Counter (OTC) drugs in many countries including the U.S. [18]. Therefore, this product should satisfy the minimum requirements set by standard agencies to provide the expected result of the quality, safety, and efficacy. The main parameter to be evaluated is the alcohol content which is the active agent responsible for the antimicrobial effectiveness [13]. The desired pH, viscosity, and hydrogen peroxide content of ABHS are also the other parameters that are related to the products’ functionality and acceptability by the users. Hence, there is a need to provide attention and control of the product’s efficacy and safety.

The products are also required to meet minimum regulatory requirements of quality standards; which may result in health risks and misleading information if violated. However, several concerns about the quality of such products have been raised by the general public, health professionals, and regulatory experts. Use of falsified ABHS may lead to a significant public health risk considering the importance of the products in preventing the spread of COVID-19 and other infections. The ABHS are considered falsified either when it contains ingredients not indicated in the approved list like methanol or when the alcohol content is below the specified limit. Exposure to the falsified ABHS can result in either systemic toxicity and, in some cases, death, due to methanol content, or vulnerability of the public to contracting and spreading COVID-19 and other infectious diseases [19]. Because of the dire demand for the products, lack of proper understanding of the impact of quality defects, or due to business orientation by manufacturers and supply chain actors, the problem might have been pronounced calling for scientific investigation and timely taking regulatory measures.

The Ethiopian Food and Drug Authority (EFDA) is legally authorized to oversee the multitude of producers to ensure that high-quality ABHS are manufactured and circulated in the marketplace. In doing so, the authority plays a critical role in protecting the users from the unwanted effects of the products. The EFDA issued temporary directive to provide regulatory flexibility to manufacturers to help meet the increased demand for these products [20]. The guidance indicated that the manufacturers should produce ABHS in accordance with WHO standards. The existing pharmaceutical industries, small-scale manufacturers, and many new companies in Ethiopia have started production and selling hand sanitizer products because of the increased demand fueled by the COVID-19 pandemic.

Therefore, in this study, the locally manufactured ABHS marketed in Addis Ababa, Ethiopia in the era of COVID-19 were evaluated for their physicochemical quality and antimicrobial efficacy against pathogenic bacteria according to the United States Pharmacopoeia (USP) and WHO standards.

## Materials and methods

### Materials

Different brands of locally manufactured ABHS were collected from the marketplaces (drug retail outlets and supermarkets) in Addis Ababa. The hand sanitizers are meant for marketing in healthcare settings and for the general public. The details of the collected hand sanitizers are described in Table 1 (with codes representing each brand). The samples were stored in their original container under ambient conditions as per the manufacturers’ recommendations until analysis. All samples were within their shelf lives during analysis.

**Table 1:** Description of locally manufactured ABHSmarketed in Addis Ababa, Ethiopia, 2021

S. no.	Product	Product’s information		
		Expiry date (month/year)	Pack size	Source
1	LPC101	10//2022	1000 ml	DROL
2	LPC102	04//2024	1000 ml	DROL
3	LPC103	04/2022	1000 ml	DROL
4	LPC104	05/2023	500 ml	DROL
5	MPC201	Not indicated	250 ml	DROL
6	MPC202	Not indicated	1000 ml	DROL
7	MPC203	04/2024	1000 ml	DROL
8	MPC204	04/2023	1000 ml	DROL
9	MPC205	Not indicated	1000 ml	DROL
10	MPC206	Not indicated	500 ml	DROL
11	SPC301	12/2023	1000 ml	DROL
12	SPC302	05/2023	1000 ml	DROL
13	SPC303	Not indicated	1000 ml	DROL
14	SPC304	04/2023	1000 ml	Supermarket
15	SPC305	03/2022	500 ml	DROL
16	SPC306	06/2023	1000 ml	DROL
17	SPC307	Not indicated	500 ml	DROL
18	SSC401	06/2022	500 ml	Supermarket
19	SSC402	05/2022	1000 ml	DROL
20	SSC403	06/2023	1000 ml	DROL
21	SSC404	12/2022	1000 ml	Supermarket
22	SSC405	01/2022	500 ml	DROL
23	SSC406	11/2022	1000 ml	Supermarket
24	SSC407	03/2022	500 ml	DROL
25	SSC408	Not indicated	250 ml	Supermarket

*DROL* Drug Retail OutLet

The chemicals, reagents and instruments used for the study include Ethanol absolute (EMSURE ACS,ISO, Reag. Ph Eur, ≥ 99.8%, Merck KGaA, Germany) which was supplied as reference standard from EFDA;; Sulfuric acid (Merck KGaA, Germany); Primary Standard Sodium Oxalate (Alfar Aesar, Great Britain); Potassium Permanganate (Blulux Laboratories P.Ltd., India); ultra-pure water (Anton Paar, Germany); Barium chloride dihydrate (BaCl2.2H2O), (LABKEMICAL,); MacConkey agar (Accumix, India); Mannitol Salt agar (SRL, India); Mueller Hinton agar (HIMEDIA, India); Nutrient broth (Accumix, India); Potassium hydroxide pellet 85% extra pure (LOBA Chemie, India); Salmonella Shigella agar (HIMEDIA, India); Sulfuric acid (LOBA Chemie, India); Violet Red Glucose agar (SRL, India); pH meter (HI 2550 Hanna I instruments); density meter (Anton Paar, Density Meter DMA 4200 M, Germany); Fourier Transform Infrared (FTIR) spectrophotometer (Bruker-Tensor-II, Germany);Centrifuge (DR AWELL, U.S.A); Incubator (BIOBASE, China); Spectrophotometer (OPTIZEN POP UV–VIS Smart Spectrophotometer, Korea); and Vortex Mixer (LAB STAC United Kingdom). *Escherichia coli, Klebsiella spp*., *Pseudomonas aeruginosa*, *Staphylococcus aureus, Salmonella spp.,* and *Shigella spp* were the test organisms used in the study.

## Methods

### Study design, area and period

A cross-sectional survey was used to collect ABHS from marketplaces (drug retail outlets and supermarkets) found in Addis Ababa. Addis Ababa is the political and commercial capital of Ethiopia with a population of over 5 million. The city is administratively divided into eleven sub-cities and 116 Woredas [21]. Because of the large market and access to facilities, pharmaceuticals and cosmetics manufacturing facilities, and distribution actors are largely concentrated around Addis Ababa and its outskirts. The sample ABHS were collected between October and November, 2021.

### Source and study population

The source population was all ABHS manufactured by local manufacturers and marketed to the community in Addis Ababa City. The ABHS which were manufactured by the selected local manufacturers and marketed in drug retail outlets and supermarkets in Addis Ababa were included in the study population.

### Eligibility criteria

- The ABHS that contained ethanol as an active ingredient, manufactured by local manufacturers, labeled with information, having usable shelf-life, and registered by EFDA were included in the study.

### Sample size and sampling techniques

At the time of data collection period, the EFDA had registered 161 hand sanitizer manufacturers nationwide and licensed their products for market; of which 124 were from Addis Ababa and its outskirts. The hand sanitizer manufacturers have different capacities and experiences in pharmaceuticals or cosmetics manufacturing. Accordingly, the manufacturers from Addis Ababa and its environs were broadly categorized into four: (i) 17 large-scale pharmaceutical and cosmetics/chemicals manufacturers; (ii) 31 medium level cosmetics and chemical manufacturers; (iii) 34 small-scale extemporaneous pharmaceuticals and supplies manufacturers; and (iv) 42 other small firms established following the COVID-19 pandemic.

Among the 124 ABHS product manufacturers, 25 (20% of 124) were included in the study by taking into consideration of sample representativeness and resource constraints, and further analyzed. Then, we proportionally allocated samples amongst the four categories/strata (4 from large scale pharmaceutical and cosmetics/chemicals, 6 from medium level cosmetics and chemical manufacturers, 7 from small scale extemporaneous pharmaceuticals and supply manufacturers, 8 from other small firms) and ABHS in each strata were selected using simple random sampling technique.

### Sample collection procedure

Once the study samples from each category had been identified, target products were purchased based on convenience from retail outlets (drug retail outlets or supermarkets) where the products were found. Each study sample with a total volume of 1000 ml (in a package size of 250 ml, 500 ml, or 1000 ml) was purchased for the study.

#### Physicochemical quality evaluation

Selected ABHS samples were tested for their physicochemical quality based on USP [22] and WHO standards [13].

### Physical examination

Physical examination was performed and recorded for colors and the presence of fragrances in sample ABHS.

#### Identification test for ethanol

An identification test for ethanol was performed as per USP 43 NF 38 [22]. A Bruker FFTIR spectroscopy equipped with Attenuated Total Reflectance sample compartment was used to generate the FTIR spectra of the sample ABHS in comparison with FTIR spectrum of the standard ethanol. The transmittance was measured concomitantly in the wavenumber range from 4000 to 400 cm^−1^ with a resolution of 4 cm^−1^. Sixteen FTIR scans were performed for each sample and reference ethanol.

#### Determination of ethanol content

The ethanol concentration (% v/v) of the ABHS samples was determined as per the USP monograph method II [22]. An oscillating transducer density meter (Anton Paar, Density Meter DMA 4200M, Germany) that has been calibrated with standard ethanol and standard water at room temperature and atmospheric pressure was used for the ethanol content level determination.

#### Determination of hydrogen peroxide strength

The hydrogen peroxide content of the samples was determined as per USP 43 NF 38 [22]. Each test was done in triplicate.

### pH determination

The pH of ABHSs was determined using calibrated digital pH meter (HI 2550 Hanna I instruments) and it was measured in triplicate.

#### Antimicrobial efficacy test

The antimicrobial efficacy study for the ABHS was conducted in the microbiology laboratory of the Bio and Emerging Technology Institute (BETin), Addis Ababa, Ethiopia.

### Test organisms

Clinical isolate bacteria like *Escherichia coli, Klebsiella spp*., *Pseudomonas aeruginosa*, and *Staphylococcus aureus* were kindly provided by the Department of Medical Laboratory, Tikur Anbessa Specialized Hospital, College of Health Sciences, Addis Ababa University whereas *Salmonella spp*. and *Shigella spp*. isolates were obtained from BETin microbiology laboratory.

### Confirmation of the test organism

For the confirmation of the test organism; gram staining and biochemical identification were conducted. The test organisms were inoculated into MacConkey agar (Accumix, India), Mannitol Salt agar (SRL, India), Salmonella Shigella agar (HIMEDIA, India), and Violet Red Glucose agar (SRL, India) and were incubated at 35–37 °C for 24 h. On the next day, a gram reaction was performed and followed by biochemical tests using their biochemical characteristics after overnight incubation (35–37 °C). The isolated test organisms were stored on storage media, kept at 2–8 °C, and used when needed. Each of the test organisms was standardized using 0.5 McFarland standard [23]. This 0.5 McFarland turbidity standard was prepared from the mixture of sulfuric acid (H_2_SO_4_) (LOBA Chemie, India) and barium chloride dihydrate (BaC_l2_ 2H_2_O) (LABKEMICAL) solution with confirmation of the mixture absorbance (0.08–0.10) density accuracy through a spectrophotometer (OPTIZEN POP UV–Vis Smart Spectrophotometer, Korea) at a wavelength of 625 nm.

### Antibacterial activity of the ABHS through agar well diffusion methods

Agar diffusion method was used to determine the susceptibility test of selected test organisms for each product sample. This agar diffusion method was done in triplicate for each sample. Standardized test organisms were swabbed into sterile Mueller Hinton agar (HIMEDIA, India) plates using sterile cotton swabs. After swabbing Mueller Hinton agar was dried; 5 equally spaced holes were bored in the agar plate with the blue tips. The 3 holes were filled with 100μL of the hand sanitizer at the same time while the other two holes were filled with an equal volume of sterile water and ampicillin suspension for negative and positive control purposes, respectively. The Mueller Hinton agar was incubated at 37° C for 24 h. The zones of inhibition of the sample products to each test organism were examined with a ruler in millimeters by considering the average of two readings that were found from a triplicate of agar diffusion test for each ABHS sample [24, 25].

### Minimum inhibition concentration (MIC) determination

The lowest concentration of an ABHS required to inhibit the growth of a known test organism in vitro was done on nutrient broth for each product sample against the selected test organisms. The minimum inhibitory concentration (MIC) was determined using broth dilution method [23] by preparing various concentrations of each product sample. Then, one milliliter from each hand sanitizer product was introduced into the tube containing equal volumes (1 mL) of nutrient broth inoculated with a standardized test organism that brings the final hand sanitizer concentrations 80, 70, 60, 50, 40, 30, 20, and 10%. A tube containing nutrient broth and bacteria without sanitizer and a tube containing the sanitizer and broth without bacteria were used as a negative and positive control, respectively. Each experiment is done in triplicates. Finally, the tubes were incubated for 18–24 h and visible growth (turbidity) was assessed. When compared with the controls, the concentration of the hand sanitizers at which no visible growth was regarded as MIC.

### Minimum bactericidal concentration (MBC) determination

The lowest concentration of a specific hand sanitizer that can kill 99.9% of a given bacterial strain was determined from the MIC tests that showed no visible growth by taking a loopful of inoculum living test organisms from the MIC tubes by streaking on fresh Mueller Hinton agar. The streaked Mueller Hinton agar plates were incubated at 37 °C for 24 h and were observed for growth. Streaked Mueller Hinton agar plates that cannot show any growth indicates a 99.9% bactericidal effect of the sanitizer at that concentration or MBC [23]. The tests were done in triplicates.

#### Quality control and data quality assurance

To maintain the quality of this project, aseptic technique was followed and all tests were performed in triplicates. Before testing, all the collected ABHS were stored as per the manufacturers’ storage conditions. All the equipment used for testing were checked for their functionality. The prepared culture media were checked for sterility by incubating five percent of the prepared media overnight and observing for the presence of any growth. The suitability of the prepared media in supporting the growth of the organisms were checked by inoculating control strains.

### Ethical clearance

Before starting the research work, ethical approval was obtained from Addis Ababa University, School of Pharmacy Ethical Review Committee (ERB/SOP/307/13/2021). This study was carried out according to the Helsinki Declaration of ethical principles for research. All the information obtained from the study about ABHSs were maintained confidential by assigning codes for the products.

### Data analysis and interpretation

Data were properly collected, analyzed, and presented using appropriate statistical tools. The data were interpreted and the results are presented as mean ± SD. Statistical analysis was performed using SPSS program version 25.

## Results

### Physicochemical quality evaluation

**Fig. 1:**
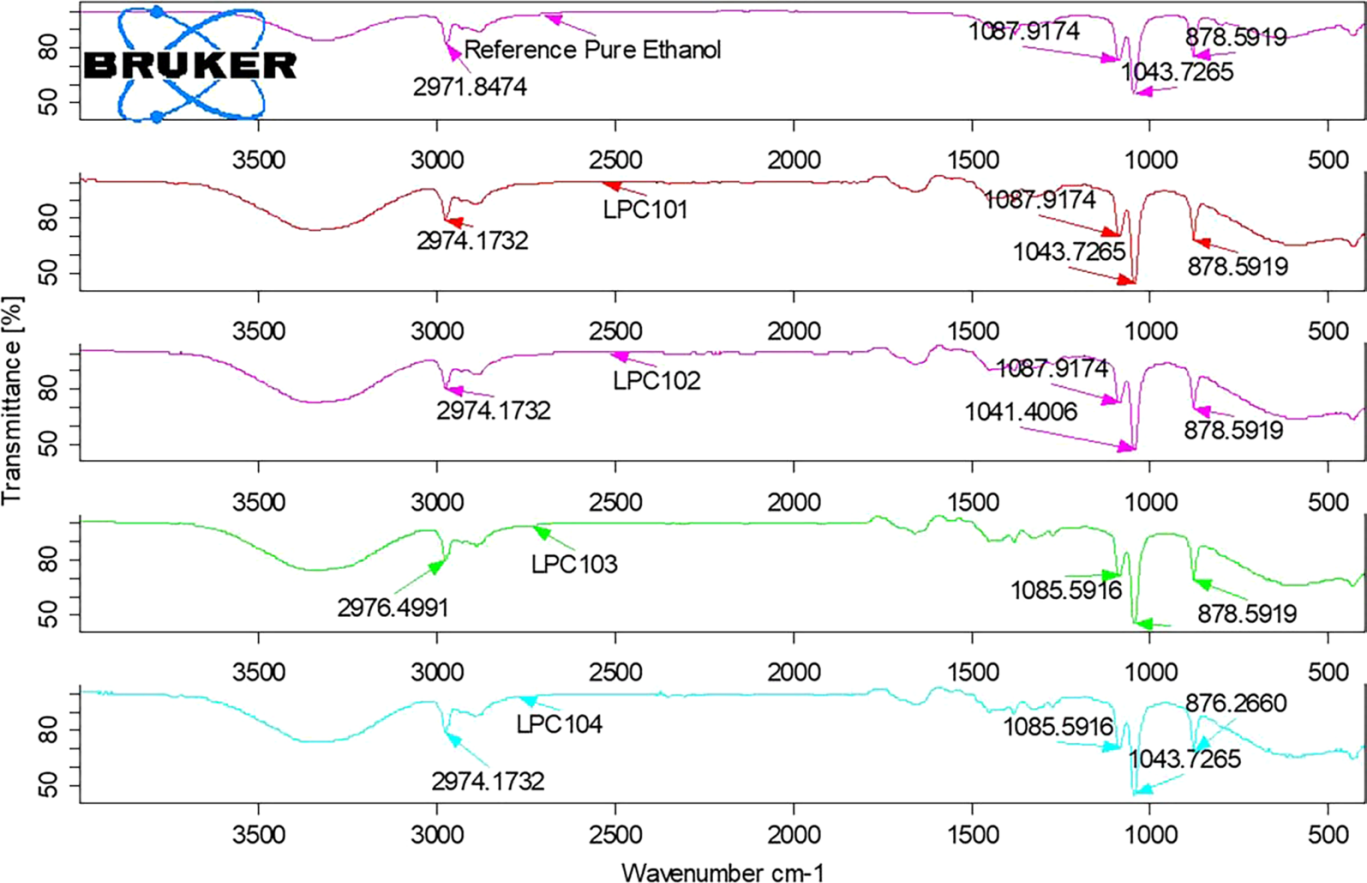
The FTIR spectrum of ethanol reference standard and tested ABHS(LPC101, LPC102, LPC103, and LPC104)

**Fig. 2:**
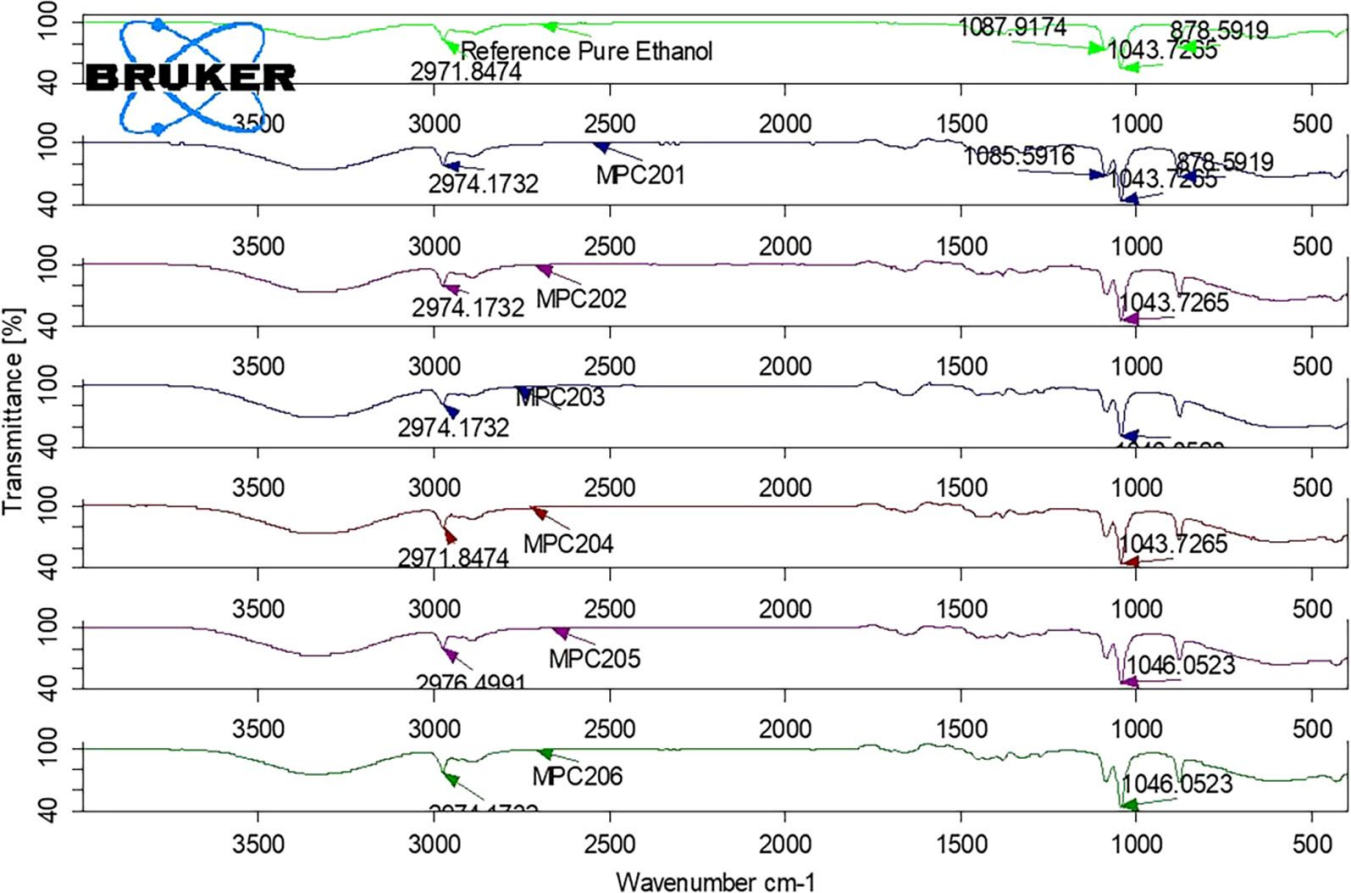
The FTIR spectrum of ethanol reference standard and tested ABHS (MPC201, MPC202, MPC203, MPC204, MPC205, and MPC206)

**Fig. 3:**
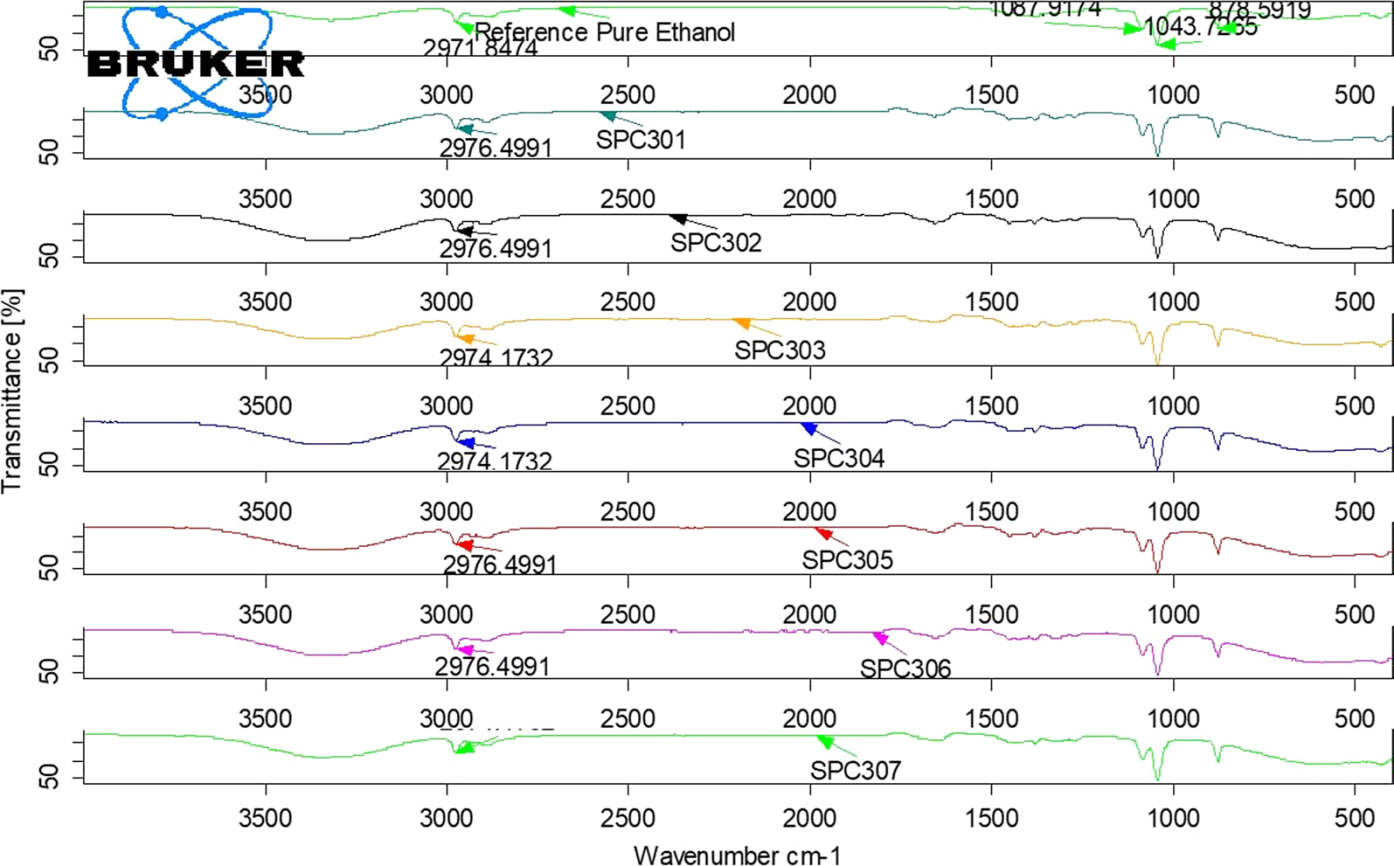
The FTIR spectrum of ethanol reference standard and tested ABHS (SPC301, SPC302, SPC303, SPC304, SPC305. SPC306, and SPC307)

**Fig. 4:**
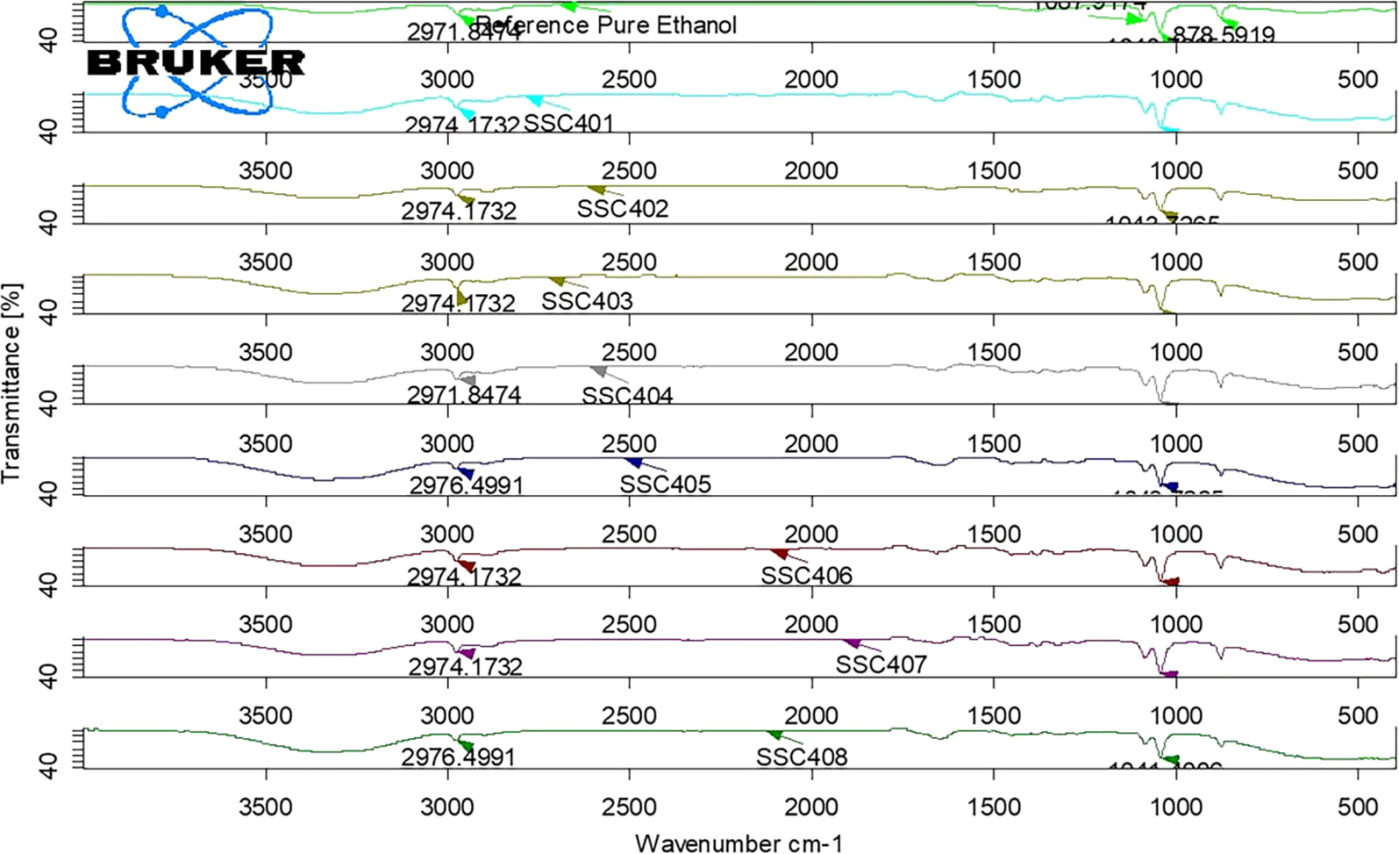
The FTIR spectrum of ethanol reference standard and tested ABHS (SSC401, SSC402, SSC403, SSC404, SSC405, SSC406, SSC407, and SSC408)

The FTIR spectra of the standard ethanol and the sample ABHS are demonstrated in Figs. 1, 2, 3 and 4. A broad absorption band was found in the standard ethanol and all the tested sample products in the region with wavenumber ranging from 3600 to 3100 cm^−1^, indicating the presence of a hydroxyl group (–OH). This peak (due to hydroxyl group (–OH)) shape and location completely different from peaks due to primary amines which specifically consist of sharp two small peaks look like a cow udder or two V-shaped hand figures, and peaks due to secondary amine absorptions are somewhat thinner and sharper than the broad and rounded absorptions produced by alcohols, indicating the presence of a hydroxyl group (–OH). Strong absorbance peaks were also observed at 878 cm^−1^ and 1043 cm^−1^. Moreover, similarity factor of FTIR spectrum of reference ethanol and tested sample FTIR spectrum was found greater than 95%.

The result of some physicochemical parameters evaluated for the collected ABHS showed that out of the 25 samples evaluated, 20 (80%) were found to be colorless solution whereas the remaining 20% exhibited certain specific colors (Table 2). LPC102 revealed the maximum ethanol concentration of 83.8%v/v. On the other hand, SSC408 had 54.4% v/v ethanol content which is the minimum value of all tested products. The least hydrogen peroxide content was found in SSC403 (0.03%v/v). A maximum pH value of 7.6 was recorded for product SPC301.

**Table 2:** Some physicochemical characteristics of ABHS marketed in Addis Ababa, Ethiopia, 2021

S. no.	Product	Test parameters				
		Ethanol conc. (%v/v) ± SD	H_2_O_2_ conc. (%v/v) ± SD	pH ± SD	Color	Fragrance
1	LPC101	78.93 ± 0.12	0.23 ± 0.00	5.40 ± 0.00	Colorless	No
2	LPC102	83.80 ± 0.10	0.16 ± 0.00	5.87 ± 0.06	Colorless	No
3	LPC103	78.60 ± 0.10	0.08 ± 0.02	4.90 ± 0.00	Colorless	No
4	LPC104	80.37 ± 0.06	0.20 ± 0.01	6.67 ± 0.06	Colorless	No
5	MPC201	80.23 ± 0.12	0.09 ± 0.01	7.13 ± 0.06	Colorless	No
6	MPC202	78.53 ± 0.12	0.25 ± 0.01	8.97 ± 0.06	Colorless	Yes
7	MPC203	69.60 ± 0.10	0.13 ± 0.00	6.03 ± 0.12	Colorless	No
8	MPC204	77.70 ± 0.10	0.38 ± 0.01	7.40 ± 0.00	Colorless	No
9	MPC205	78.83 ± 0.06	0.31 ± 0.01	5.80 ± 0.00	Colorless	No
10	MPC206	82.33 ± 0.06	0.24 ± 0.01	5.43 ± 0.06	Colorless	No
11	SPC301	77.03 ± 0.06	0.29 ± 0.01	7.60 ± 0.10	Colorless	No
12	SPC302	69.67 ± 0.06	0.22 ± 0.01	6.87 ± 0.06	Light orange	No
13	SPC303	80.73 ± 0.06	0.24 ± 0.01	6.53 ± 0.25	Light green	No
14	SPC304	77.47 ± 0.06	0.22 ± 0.02	6.33 ± 0.06	Light red	No
15	SPC305	78.57 ± 0.06	0.29 ± 0.01	6.77 ± 0.06	Colorless	No
16	SPC306	72.57 ± 0.06	0.19 ± 0.01	5.77 ± 0.06	Light green	Yes
17	SPC307	78.60 ± 0.10	0.27 ± 0.01	6.23 ± 0.06	Colorless	No
18	SSC401	72.70 ± 0.10	0.23 ± 0.00	6.50 ± 0.00	Colorless	No
19	SSC402	82.63 ± 0.12	0.04 ± 0.01	6.83 ± 0.12	Colorless	No
20	SSC403	72.63 ± 0.12	0.03 ± 0.01	6.57 ± 0.06	Colorless	No
21	SSC404	80.03 ± 0.12	0.16 ± 0.02	7.33 ± 0.06	Light yellow	No
22	SSC405	56.80 ± 0.17	0.13 ± 0.00	8.57 ± 0.06	Colorless	No
23	SSC406	74.40 ± 0.10	0.24 ± 0.00	6.40 ± 0.00	Colorless	No
24	SSC407	77.33 ± 0.12	0.14 ± 0.01	5.57 ± 0.06	Colorless	No
25	SSC408	54.43 ± 0.15	0.09 ± 0.02	8.47 ± 0.06	Green	No

#### Antimicrobial evaluation

All of the test organisms were confirmed for their credentials with different biochemical tests. The antimicrobial effectiveness was assessed by measuring the zone of inhibition against the specific test bacteria. Maximum inhibition was seen in LPC 103 and SSC 407 sanitizer against *Shigella spp*. and *Salmonella spp.,* respectively i.e., 15 mm. The minimum inhibition was seen in MPC 204 against *Escherichia coli* i.e., 3 mm (Table 3).

**Table 3:** Zones of inhibition of selected ABHS against test organisms

Hand sanitizer	Ethanol conc. (%v/v) ± SD	Zones of Inhibition (mm)					
		*Escherichia coli*	*Pseudomonas aeruginosa*	*Staphylococcus aureus*	*Salmonella spp.*	*Shigella spp.*	*Klebsiella Spp*
LPC 101	78.93 ± 0.12	10	12	11	10	12	6
LPC 102	83.80 ± 0.10	6	4	5	6	12	5
LPC 103	78.60 ± 0.10	11	11	9	13	15	6
LPC 104	80.37 ± 0.06	4	13	6	10	10	8
MPC 201	80.23 ± 0.12	8	8	4	4	4	4
MPC 202	78.53 ± 0.12	10	8	6	5	6	7
MPC 203	69.60 ± 0.10	9	9	5	5	4	5
MPC 204	77.70 ± 0.10	3	10	10	10	14	12
MPC 205	78.83 ± 0.06	10	12	7	10	10	8
MPC 206	82.33 ± 0.06	9	4	4	7	5	4
SPC 301	77.03 ± 0.06	8	11	7	8	9	6
SPC 302	69.67 ± 0.06	10	8	5	7	5	6
SPC 303	80.73 ± 0.06	11	10	8	8	12	5
SPC 304	77.47 ± 0.06	10	10	5	9	4	5
SPC 305	78.57 ± 0.06	10	5	8	6	5	8
SPC 306	72.57 ± 0.06	9	8	5	5	6	4
SPC 307	78.60 ± 0.10	12	9	10	4	4	6
SSC 401	72.70 ± 0.10	10	10	10	10	9	4
SSC 402	82.63 ± 0.12	5	11	5	4	5	5
SSC 403	72.63 ± 0.12	4	14	9	10	12	6
SSC 404	80.03 ± 0.12	7	5	5	5	4	5
SSC 405	56.80 ± 0.17	5	4	9	4	5	4
SSC 406	74.40 ± 0.10	7	13	10	9	8	9
SSC 407	77.33 ± 0.12	10	7	12	15	5	4
SSC 408	54.43 ± 0.15	9	13	13	10	4	7

Table 4 shows the Minimum Inhibitory Concentration (MIC) of the tested ABHS. The results revealed all hand sanitizer products displayed antibacterial activity against all of the test bacteria at a minimum concentration from 10 to 80%. Thus, LPC 101 hand sanitizer showed a 10% minimum inhibitory concentration against *E. coli*, *P. aeruginosa,* and *Klebsiella spp*. Similarly, LPC 102 and SPC 305 hand sanitizers exhibited 10% MIC against *Staphylococcus aureus.* Congruently, 10% MIC was also observed by MPC 203 hand sanitizer against *Escherichia coli.* The MIC of the majority of hand sanitizers lied 10–50% nearly for all of the tested bacteria.

**Table 4:** Percent of minimum inhibitory concentration (MIC) of selected ABHS against test organisms

Hand sanitizer	Ethanol conc. (%v/v) ± SD	Minimum inhibitory concentration (MIC (%))					
		*Escherichia coli*	*Pseudomonas aeruginosa*	*Staphylococcus aureus*	*Salmonella spp.*	*Shigella spp.*	*Klebsiella Spp*
LPC 101	78.93 ± 0.12	10	10	70	30	20	10
LPC 102	83.80 ± 0.10	40	30	10	40	50	40
LPC 103	78.60 ± 0.10	50	80	20	40	60	40
LPC 104	80.37 ± 0.06	30	30	40	30	50	50
MPC 201	80.23 ± 0.12	30	40	50	60	60	30
MPC 202	78.53 ± 0.12	50	20	30	30	30	20
MPC 203	69.60 ± 0.10	10	30	40	40	30	20
MPC 204	77.70 ± 0.10	20	20	20	30	30	30
MPC 205	78.83 ± 0.06	20	30	40	40	40	20
MPC 206	82.33 ± 0.06	60	50	60	60	70	50
SPC 301	77.03 ± 0.06	20	30	50	40	50	40
SPC 302	69.67 ± 0.06	20	20	60	60	50	30
SPC 303	80.73 ± 0.06	20	20	80	50	50	50
SPC 304	77.47 ± 0.06	60	80	80	60	60	60
SPC 305	78.57 ± 0.06	30	30	10	50	80	70
SPC 306	72.57 ± 0.06	60	50	30	40	50	60
SPC 307	78.60 ± 0.10	30	50	60	40	30	40
SSC 401	72.70 ± 0.10	30	20	70	30	30	40
SSC 402	82.63 ± 0.12	30	30	60	60	60	60
SSC 403	72.63 ± 0.12	40	50	50	60	50	50
SSC 404	80.03 ± 0.12	40	40	60	50	50	50
SSC 405	56.80 ± 0.17	50	60	60	40	30	70
SSC 406	74.40 ± 0.10	60	50	70	50	50	50
SSC 407	77.33 ± 0.12	70	50	70	50	70	70
SSC 408	54.43 ± 0.15	60	60	60	30	50	50

The minimum bactericidal activity of the hand sanitizers against test bacteria was found to be in the range of 20% to 80% (Table 5). From the assessed twenty-five hand sanitizers, seven of them showed 20% bactericidal activity against test bacteria. Of which LPC 102, MPC 202, and MPC 204 hand sanitizers exhibited below 50% bactericidal activity against all of the test bacteria.

**Table 5:** Percent of minimum bactericidal concentration (MBC) of selected ABHS against test organisms

Hand sanitizer	Ethanol conc. (%v/v) ± SD	Minimum bactericidal concentration (MBC (%))					
		*Escherichia coli*	*Pseudomonas aeruginosa*	*Staphylococcus aureus*	*Salmonella spp.*	*Shigella spp.*	*Klebsiella Spp*
LPC 101	78.93 ± 0.12	20	20	80	40	30	20
LPC 102	83.80 ± 0.10	30	40	30	30	40	30
LPC 103	78.60 ± 0.10	40	70	30	50	50	50
LPC 104	80.37 ± 0.06	40	40	50	40	60	60
MPC 201	80.23 ± 0.12	40	50	60	50	50	40
MPC 202	78.53 ± 0.12	40	30	40	20	40	30
MPC 203	69.60 ± 0.10	20	40	50	50	40	30
MPC 204	77.70 ± 0.10	30	30	30	40	40	40
MPC 205	78.83 ± 0.06	30	40	50	50	50	30
MPC 206	82.33 ± 0.06	50	40	70	50	60	40
SPC 301	77.03 ± 0.06	30	40	60	50	60	50
SPC 302	69.67 ± 0.06	30	30	70	50	40	40
SPC 303	80.73 ± 0.06	30	30	70	40	40	40
SPC 304	77.47 ± 0.06	50	70	70	50	50	50
SPC 305	78.57 ± 0.06	40	40	20	40	70	60
SPC 306	72.57 ± 0.06	70	40	40	50	60	70
SPC 307	78.60 ± 0.10	40	40	70	50	20	30
SSC 401	72.70 ± 0.10	40	30	60	40	20	50
SSC 402	82.63 ± 0.12	20	20	50	50	50	50
SSC 403	72.63 ± 0.12	30	40	60	50	60	60
SSC 404	80.03 ± 0.12	30	30	50	40	40	40
SSC 405	56.80 ± 0.17	40	50	70	50	40	60
SSC 406	74.40 ± 0.10	50	40	60	40	40	40
SSC 407	77.33 ± 0.12	60	40	60	40	60	60
SSC 408	54.43 ± 0.15	50	50	50	20	40	40

## Discussion

Promoting good hygiene in healthcare facilities and communities is important to avoid pathogenic diseases [26]. Hand hygiene practice is an essential part of daily life which is the simplest and least expensive measure proven to be effective in preventing COVID-19 and other infections to keep humans healthy [6, 7]. Among the range of strategies proposed for the promotion and improvement of hand hygiene, use of hand sanitizers is well advocated as it offers a convenient, effective, and relatively low-cost alternative, especially for developing countries [27–29].

The WHO recommended use of ABHS with ethyl alcohol at a concentration of 80% v/v for optimal antimicrobial efficacy [10]. If failed to meet minimum quality standards, hand sanitizer can be ineffective (misleading users due to perceived effectiveness and aggravating the spread of COVID-19 and other infections) and also cause public health risks. There are also many risks associated with low quality hand sanitizers which include harm to healthcare providers, patients, and the general public. Unless these quality issues are addressed and managed appropriately, the risks outweigh the benefits of these products.

The current study attempted to evaluate the physicochemical and antimicrobial efficacy of sample ABHS marketed in Addis Ababa sourced from different local manufacturers following the outbreak of the current pandemic COVID-19. All the test products were formulated as per WHO formulation [13] that contains ethanol, glycerol, and hydrogen peroxide ingredients with an anticipated concentration of 80% v/v, 1.45% v/v, and 0.125 v/v%, respectively in the final product. The reliability of products labelling information was checked with physicochemical analysis,

The FTIR spectra of all the tested sample products showed the presence of ethanol in the formulations as the characteristic peaks of ethanol are indicated in the Figs. 1, 2, 3 and 4. The appearance of strong absorbance peaks at 878 cm^−1^ (C–C–O symmetric stretch) and 1043 cm^−1^ (C–O stretch of primary alcohol) could serve as the signature FTIR characteristics for ethanol [22, 27]. The result revealed the matching of alcohol type indicated in the labels with the analysis outcome. It’s essential to check the presence of the claimed alcohol in the formulation since hand sanitizers devoid of the labeled ingredient may be circulated in the market due to their current high demand. A study conducted in Nairobi showed about 14.9% of the tested 74 samples had methanol, instead of ethanol, as the main component of ABHS [30]. Another study in Johannesburg area revealed that 3 of the 94 different hand sanitizer products were found to contain no alcohol [31]. Such circulation of falsified hand sanitizer products in the market compromises the control of infection transmission and may expose users to the undesired effects. Consumers, for example, may experience poisoning when exposed to hand sanitizer containing methanol which is not an acceptable formulation ingredient. Nausea, vomiting, blindness, seizures, coma, damage to the nervous system or death may be resulted from methanol exposure that seek immediate treatment for reversal of such toxic effects [32].

Considering the physical appearance of the tested products, all samples were in solution form as stated on their label and 20% exhibited distinct colors such as light green, light yellow, and red whereas the remaining 80% were found to be colorless. Moreover, two products contained fragrance in the formulations, as indicated on their labels. Fragrances and coloring agents are commonly incorporated in formulations to increase the acceptability of the product and for product identification. But, it is clearly indicated that such addition of fragrances and colorants is not recommended due to the potential risk of allergic reactions and might increase the risk of ingestion by children [13, 33, 34]. In addition to this, the antimicrobial effectiveness of the products may be compromised by these agents which otherwise their influence should be justified with tests [13, 17]. It is also possible that the inclusion of additional ingredients, particularly when untested, would affect product efficacy, stability, and safety [4, 35]. However, based on the findings from the study, there was no correlation between the color of the hand sanitizer samples with the other attributes measured or the FTIR spectrum.

Ethanol is the main active agent in the formulation that is responsible for the lethality of microorganisms. As the efficacy of alcohol is dependent on its concentration, the accurate determination of alcohol content of ABHS may act as a surrogate for efficacy [30, 35]. The limit of ethanol content to comply with the requirement is stated to be within ± 5% variation (75–85%) from the claimed potency (80% v/v) [13]. Density measurement was explored as an approach for estimation of the ethanol content [36]. The current evaluation result depicted that those 8 products (32%) failed to meet the requirements and all were found to contain lower content for ethanol (< 75% v/v). The maximum variation was noted for SSC408 with only 54% v/v ethanol content. Five out of the eight products that failed the test for ethanol content were from the small-scale manufacturer’s category (i.e. SSC) which indicates the need for close control of such companies by the regulatory body. Such quality defect of the hand sanitizers may lead to poor hand hygiene and contributing to healthcare-associated infections as the study samples were also meant for use in healthcare settings.A similar study done in Johannesburg resulted in 37 (41%) products containing less than 60% v/v alcohol [31]. Additional literature have also reported the circulation of substandard ABHS in various market places [31, 37–40]. The concentration of ethanol beyond the specified limit leads to lack of antimicrobial role and compromise the hand hygiene promotion program [13].

Due to the increasing consumer demand, these products could become easy targets of fraud or counterfeiting by bulking the preparation by diluting the alcohol content with water or cheaper substitutes like methanol which end up with a less functional product [17].

Moreover, the influence of other formulation ingredients on ABHS efficacy, safety, and usage should be taken into consideration. Hydrogen peroxide is among the ingredients which are added to avoiding spore-forming organisms in the product [13]. Spore forming organisms may result from the raw materials such as water and the packaging bottles or during the production process. The limit of acceptance, according to USP specification, to hydrogen peroxide topical solution is found to be in the range of 90–110% of the claimed potency. Only three products (MPC203, SSC405, and SSC407) gave a satisfactory result for the hydrogen peroxide content test (0.112–0.137% v/v) [22]. The maximum and minimum concentration of hydrogen peroxide was found to be 0.38% v/v and 0.03% v/v, respectively. The availability of this ingredient beyond the required limit affects either the performance of the product or creates discomfort to the users. Despite its importance in the formulation of ABHS, the presence of hydrogen peroxide in the product at higher concentration is associated with toxicity. The risk may range from mild irritation of the eyes and skin when used externally to irritation of the inside of the mouth and the gastrointestinal tract, and air embolism when ingested [41].

The optimum pH value of hand sanitizers is important for the effectiveness of the product as well as for its suitability during application on hands. The incorporation of ingredients beyond the defined concentration or other ingredients (such as colorants) may affect the pH of the final product. The tested products showed a pH range between 4.90 and 8.97. Normally, skin pH range between 5.4 and 5.9, [42, 43] and this neutral pH is generally accepted for cosmetic products. Only 6 products (24%) lay in this pH range and the majority (72%) had exhibited higher pH values. Such high pH levels might be resulted from the nature of ingredients incorporated in the formulation of ABHS. It is important to consider skin pH during the formulation of dermatological products like hand sanitizers so that the product will not cause skin dryness or irritation and brings soft and smooth skin. Overall, considering the tests outlined in Table 2, only one product (SSC407) complied with all the physicochemical tests.

Many studies have been conducted to assess the quality and antimicrobial effectiveness of hand sanitizers elsewhere and failure to meet the quality standard has been reported for some products [16, 38, 44, 45]. Following the public health emergency due to COVID-19, the EFDA has licensed more than 100 manufacturers for the production of ABHS to meet the growing demand for this product in the country. Even though these products are considered as drugs [18], interested companies without the required professionals are allowed to engage in manufacturing the sanitizers to address the supply shortage. In addition, some beverage firms have reconfigured their operations to produce hand sanitizer products. Such involvement of individuals without adequate knowledge and experience for similar products may contribute to the poor quality of products and there are regulatory requirements to be known.

Because of lack of manufacturers’ understanding or due to business orientation, quality defects are often reported by regulatory authorities and individual users which may endanger users’ safety. Moreover, there are several hand sanitizers sold to the Ethiopian market with labels on their package that claim that the hand rub can kill 99.9% of germs without generating evidence. This problem may be further intensified in light of a limited regulatory capacity to conduct regular inspection and quality surveillance.

Despite the claims of efficacy and 99.9% bacterial reduction by hand sanitizer manufacturers, there still exists a need for verification of these claims. The present study also evaluated the sample products for their antibacterial efficacy. All the ABHS displayed bactericidal activity against all the selected test organisms at a concentration of range from 20 to 80%. Subsequently, the highest bactericidal effect was observed against *S. aureus* with 80% activities. This is in line with the findings of a similar study conducted by Otokunefor and Princewill [46]. Contrarily, other studies [47, 48] showed that efficacy on *E.coli* was higher compared to the other pathogens.

LPC 101 ABHS had the highest bactericidal activity against *S. aureus*. Subsequently, SPC 304 ABHS was the most effective hand sanitizer against all the tested bacteria with a range of 50—70% bactericidal activities. Consistently, research finding has shown hand sanitizers to have antimicrobial effects against bacteria such as *S. aureus*, *E. coli*, *Pseudomonas spp*., and *Klebsiella Spp.* [49].

The minimum bactericidal activity was observed in most of hand sanitizers based on their respective concentrations and various bacterial strains. Correspondingly, MPC 202 exhibited the lowest bactericidal activity (20 to 40%) against all of the test bacteria. In line with this, a study conducted by Otokunefor and Princewill [46] revealed that 25% was the minimum concentration of bacterial inhibition which below 25% was the minimum bactericidal concentration. In contrast to the current study, hand sanitizers were found to be not efficacious against test bacteria in another study [48]. Minimum bactericidal activities could be due to the relatively decreased concentration of ethanol in hand sanitizer as the efficacy of alcohol-based hand sanitizer is affected mainly by the type and content of alcohol used. Moreover, the minimum bactericidal effect could be due to poor or extended storage of the hand sanitizer which could lead to increased temperature causing evaporation of the active ingredient. Due to this, not all sanitizers are equally effective in eliminating all microorganisms [50, 51]. Provided that there is rational use of quality ABHS available in health facilities and the communities, a decrease in the incidence of multidrug-resistant bacterial and viral isolates and patient colonization will be observed [10].

Considering the pandemic COVID-19 and other infections, consumers shall be vigilant about which hand sanitizers they use. The findings of the current study revealed the spectrum/status of locally manufactured ABHS quality and antimicrobial efficacy which can help various stakeholders to implement timely interventional strategies on parameters in which defects were observed through proper public education, and engagement of key stakeholders. It also provides the regulatory body (EFDA) with objective evidence to take appropriate regulatory measures.

## Limitations of the study

This study has some limitations. The antimicrobial efficacy test was determined only for bacteria through the ABHS is also known for its effect on enveloped viruses like SARS-CoV-2 laboratory setup constraint. In addition, the study is limited to hand sanitizers manufactured as per the WHO formulation 1 (i.e. solution form). The study also failed to determine the methanol limit for the ABHS due to the unavailability of a validated method and gas chromatography for methanol content determination. However, to the best of our knowledge, this study is the first to comprehensively evaluate the physicochemical quality and efficacy of ABHS in the market obtained from local manufacturers.

## Conclusion

Quality problems of the ABHS in the market were observed especially for the hydrogen peroxide and ethanol content. About one-third of the tested products failed to satisfy the WHO requirement for the ethanol content. Moreover, the majority of the products showed higher pH values than the recommended range.

Most of the ABHS exhibited antibacterial activity against all of the test bacteria at a minimum concentration from 10 to 80%. Correspondingly, the range of MBC lies from 20 to 80%. Though, the products did not show 99.9% bacteriostatic or bactericidal activities as claimed.

Hand hygiene is recognized as the best and most cost-effective way to prevent the spread of infectious diseases, and this study contributes to the implementation of appropriate actions by the concerned stakeholders regarding the quality and efficacy of ABHS circulating in the Addis Ababa market.

## Recommendations

Hand sanitizers have become an essential product in hospitals and communities in day-to-day life. They have gained much popularity and have become a highly accepted form of personal hygiene because of their effectiveness and ease of use. Assuring the quality of these products will enhance the compliance of healthcare providers and other individuals with these products and contributes to the containment of COVID-19 and other infections.

The quality of ABHS in the local market should be given attention and addressed carefully even after the end of the current pandemic, COVID-19.

As the hand sanitizer products are considered over-the-counter (OTC) drugs, periodical inspection/evaluation of these products should be in place by the responsible organizations such as regulatory authority (EFDA), research, and academic institutions to make sure that quality products are reaching the market. The regulatory body should take the leading role in controlling the ABHS products at every stage of their lifecycle, including manufacturing and distribution, to ensure that the products are safe and effective. Moreover, the presence of methanol in the ABHS and their compliance with the specifications have to be assessed to protect the users from unwanted effects.

Despite the claims of efficacy and 99.9% bacterial reduction by hand sanitizer manufacturers, there still exists a need for verification of these claims.

## Data Availability

The datasets used for this publication can be obtained from the corresponding author on reasonable request.
